# Outstanding Reviewers for *Nanoscale Advances* in 2020

**DOI:** 10.1039/d1na90040d

**Published:** 2021-05-25

## Abstract

We would like to take this opportunity to highlight the Outstanding Reviewers for *Nanoscale Advances* in 2020, as selected by the editorial team for their significant contribution to the journal.
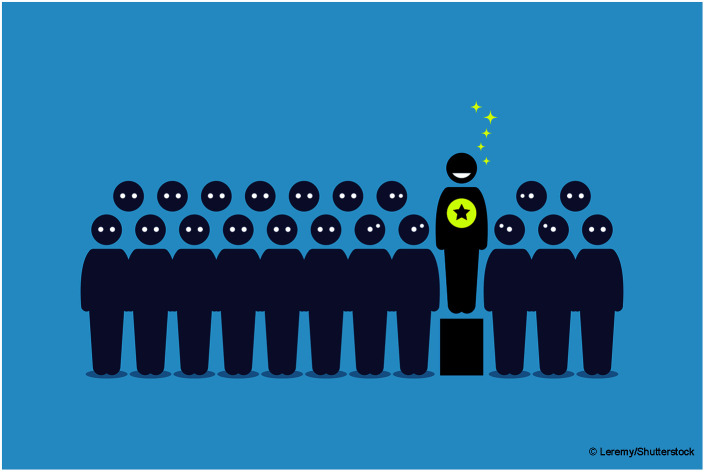

We would like to take this opportunity to thank all of *Nanoscale Advances*’ reviewers, and in particular highlight the Outstanding Reviewers for the journal in 2020, as selected by the editorial team for their significant contribution to *Nanoscale Advances*. We announce our Outstanding Reviewers annually and each receives a certificate to give recognition for their contribution. The reviewers have been chosen based on the number, timeliness and quality of the reports completed over the last 12 months.

 

Dr Carlo Casari

Milan Polytechnic

ORCID: 0000-0001-9144-6822

 

Dr Ricardo Garcia

CSIC

ORCID: 0000-0002-7115-1928

 

Wenwu Xu

Ningbo University

ORCID: 0000-0002-0651-9562

 

Dr Yifu Zhang

Dalian University of Technology

ORCID: 0000-0003-2546-9502

 

Dr Yuebing Zheng

University of Texas at Austin

ORCID: 0000-0002-9168-9477

 

We would also like to thank the *Nanoscale Advances* Editorial Board and Advisory Board and the nanoscience community for their continued support of the journal, as authors, reviewers and readers.

 

Dr Anna Rulka, Executive Editor

## Supplementary Material

